# Supplementary material for: Polymorphisms in Pfkelch13 domains before and after the introduction of artemisinin-based combination therapy in Southwest Nigeria
Source: PLoS One. 2025 Mar 31;20(3):e0316479. doi: 10.1371/journal.pone.0316479 (PMC11957316; doi:10.1371/journal.pone.0316479)

Samples: 12580
Bases: 927
Average spacing: 14

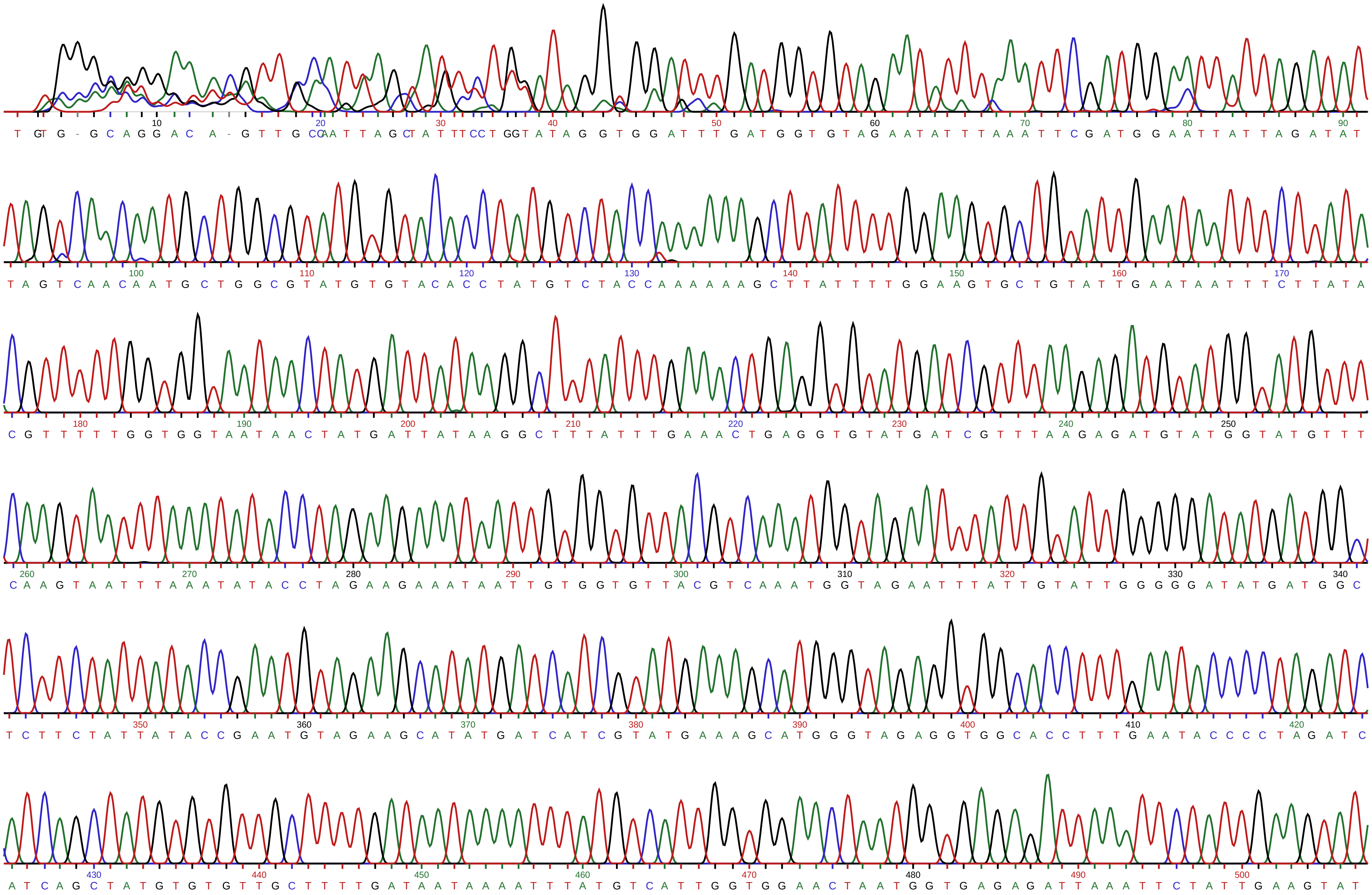

Samples: 12580
Bases: 927
Average spacing: 14

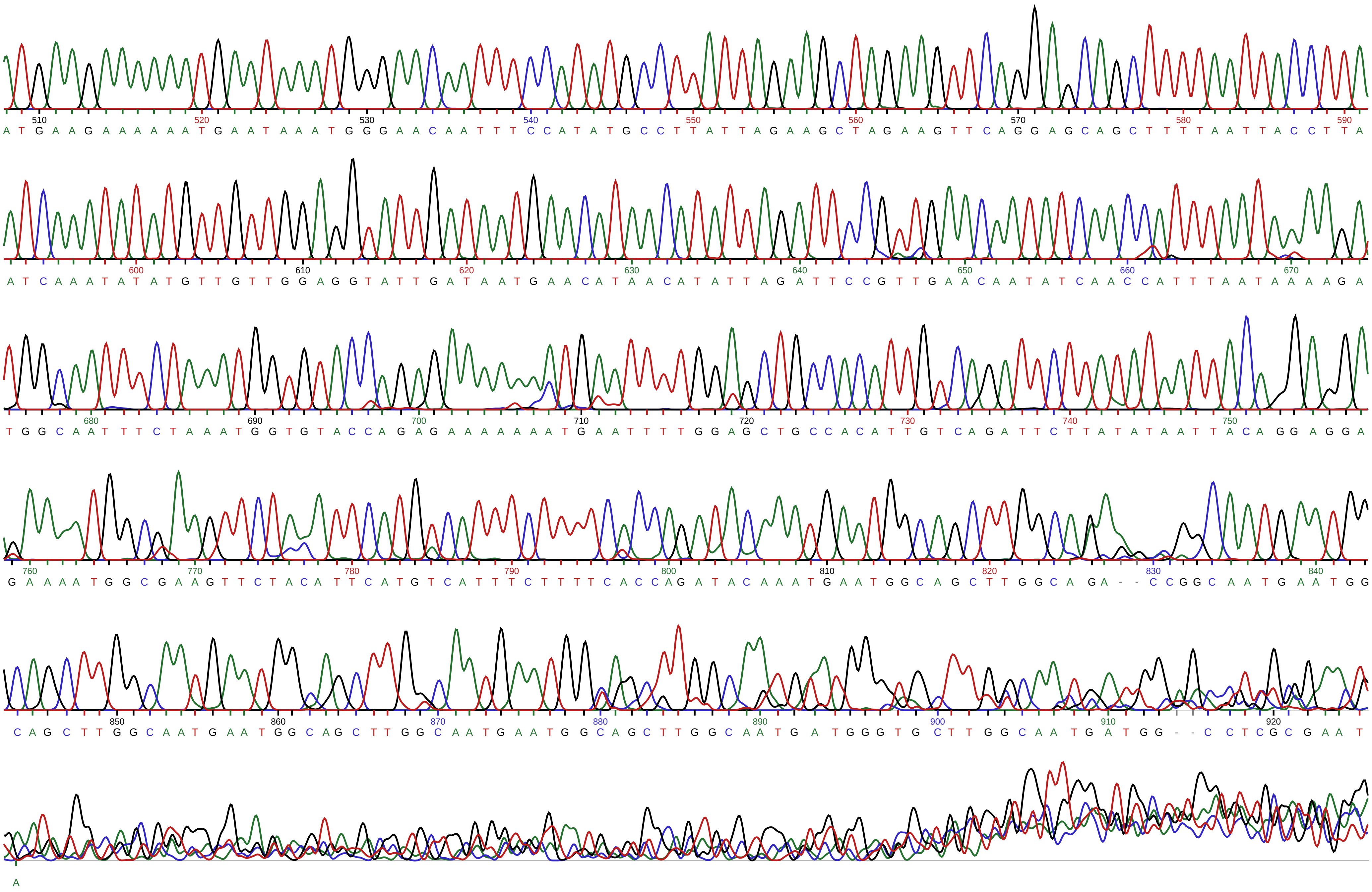

Samples:	12580
Bases:	927
Average spacing:	14

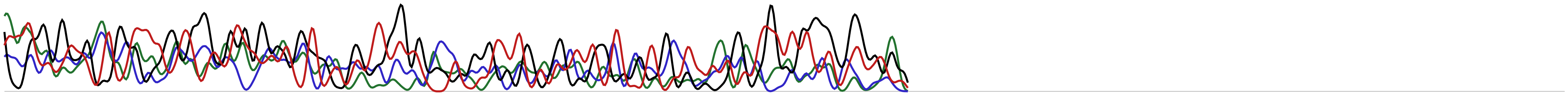

Supplement: Supporting information 2 — (ZIP) [file pone.0316479.s002.zip › 051KNIFW_PREMIX_Plate_CORKELCH_G02.pdf]
